# Efficacy of Resveratrol Supplementation on Glucose and Lipid Metabolism: A Meta-Analysis and Systematic Review

**DOI:** 10.3389/fphys.2022.795980

**Published:** 2022-03-31

**Authors:** Qian Zhou, Yanmei Wang, Xuke Han, Shunlian Fu, Chan Zhu, Qiu Chen

**Affiliations:** Hospital of Chengdu University of Traditional Chinese Medicine, Chengdu, China

**Keywords:** resveratrol, meta-analysis, lipid, T2DM, obese

## Abstract

**Background:**

Lipids are ubiquitous metabolites with diverse functions. Excessive lipid accumulation can trigger lipid redistribution among metabolic organs such as adipose, liver and muscle, thus altering the lipid metabolism. It has been revealed that disturbed lipid metabolism would cause multiple disease complications and is highly correlated with human morbidity. Resveratrol (RSV), a phytoestrogen with antioxidant, can modulate insulin resistance and lipid profile. Recently, research on RSV supplementation to improve glucose and lipid metabolism has been controversial. A meta-analysis may provide a scientific reference for the relationship between lipid metabolism and RSV supplementation.

**Methods and Analysis:**

We searched the PubMed, Cochrane Library, Web of Science, and Embase databases from inception to October 2021 using relevant keywords. A comprehensive search for randomized controlled trials (RCTs) was performed. For calculating pooled effects, continuous data were pooled by mean difference (MD) and 95% confidence interval (CI). Adopting the method of inverse-variance with a random-effect, all related statistical analyses were performed using the Rev Man V.5.3 and STATA V.15 software.

**Results:**

A total of 25 articles were incorporated into the final meta-analysis after removal of duplicates by checking titles and abstracts and excluding non-relevant articles. The selected articles had a total of 1,171 participants, including 578 in the placebo group and 593 in the intervention group. According to the current meta-analysis, which demonstrated that there was a significant decrease in waist circumference (SMD = –0.36; 95% CI: –0.59, –0.14; *P* = 0.002; *I*^2^ = 88%), hemoglobin A1c (–0.48; –0.69, –0.27; *P* ≤ 0.001; *I*^2^ = 94%), total cholesterol (–0.15; –0.3, –0.01; *P* = 0.003; *I*^2^ = 94%), low density lipoprotein cholesterol (–0.42; –0.57, –0.27; *P* ≤ 0.001; *I*^2^ = 92%), high density lipoprotein cholesterol (0.16; –0.31, –0.02; *P* = 0.03; *I*^2^ = 81%) following resveratrol administration.

**Conclusion:**

These results suggest that RSV has a dramatic impact on regulating lipid and glucose metabolism, and the major clinical value of resveratrol intake is for obese and diabetic patients. We hope that this study could provide more options for clinicians using RSV. Furthermore, in the future, large-scale and well-designed trials will be warranted to confirm these results.

**Systematic Review Registration:**

Website [https://www.crd.york.ac.uk/prospero/#recordDetails], identifier [CRD42021244904].

## Introduction

Lipids are ubiquitous metabolites with diverse functions. Excessive lipid accumulation can trigger lipid redistribution among metabolic organs such as adipose, liver and muscle, thus altering the lipid metabolism (Zhao et al., 2020). Disturbed lipid metabolism will cause multiple disease complications and is highly correlated with human morbidity (Cao et al., 2020). Even some reports have indicated that lipid redistribution was tightly associated with progression of various cancers (Liu et al., 2018), and these discoveries might be significant for treatment of antileukemic and epigenetic effects (Grønningsæter et al., 2019). Regulation of lipid metabolism is essential for maintenance of whole-body metabolic and energy homeostasis (Warne et al., 2011).

Resveratrol (RSV) is a phytoestrogen with antioxidant and can modulate insulin resistance and lipid profile (Szkudelska and Szkudelski, 2010). In previous studies, RSV has been suggested to improve motor function, extension of life span and well loss in weight in animal models (Alberdi et al., 2011; Wang et al., 2014), such as such as diminishing the deposits of white adipose tissue (WAT) and reducing total body fat (Alberdi et al., 2011). However, it was reported that for obese men, high-dose resveratrol (hRSV) used for four weeks had no effect on ectopic or visceral fat content and lipid oxidation rates (Poulsen et al., 2013). Also, RSV is a plant-derived nutritional supplement shown to have antidiabetic properties in many animals models (Sahebkar, 2013; Sahebkar et al., 2015; Tabrizi et al., 2020). In summary, the research on RSV supplementation improving glucose and lipid metabolism remains controversial.

Systematic review and meta-analysis were performed to summarize the published clinical trials to date, and we tried to incorporate the evidence as a new model for revaluating the effect of RSV on glucose and lipid metabolism more comprehensively. The results of data-analysis further define the relationship between lipid metabolism and RSV supplementation, clarifying the contribution of RSV in lipid-related components and elucidating the comparative causal role of lipid-related components by RSV supplementation.

### Objectives

(1) To provide insights into the relationship between lipid metabolism and RSV supplementation; (2) to identify resveratrol contributions in lipid-related components; (3) to elucidate the comparative causal role of lipid-related components by RSV supplementation.

### Methods and Analysis

Meta-analysis, a statistical procedure for systematic statistical synthesis of data from independent studies, is the primary source of concise up-to-date information (Nakagawa et al., 2017). We performed a meta-analysis in accordance with the methodology described in Paterson et al. (2001) and included quantitative, qualitative, and mixed-method studies (Moore et al., 2015). Preferred Reporting Items for Systematic Reviews and Meta-Analyses protocols (PRISMA-P) were followed to perform a systematic review (No author list, 2016). Methods were designed based on PRISMA (Page et al., 2021), a proposal for reporting (Stroup et al., 2000), and Cochrane Collaboration Handbook.

### Search Strategy

We searched the PubMed, Cochrane Library, Web of Science, Google Scholar, and Embase databases from inception to October 2021 using relevant keywords, and a comprehensive search for human randomized controlled trials (RCTs) was also performed. All ongoing RCTs were searched in the International Standard Randomized Controlled Trial Number register (ISRCTN), WHO International Clinical Trials Registry Platform (ICTRP), and Clinical Trials. There was a systemic search for relevant literature and exploration of the association between treatment with resveratrol and biological indexes. We used combinations of the following keywords and MeSH terms for the literature search: intervention (“resveratrol” or “resveratrols” and “supplementation” or “intake” or “use”) and outcome (“body weight” or “body mass index” or “waist circumference” or “Hemoglobin A1c” or “HOMA index” or “Insulin” or “glucose” or “fat percentage total cholesterol” or “triglyceride” or “low density lipoprotein cholesterol” or “high density lipoprotein cholesterol” or “leptin” or “adiponectin”).

### Selection Exclusion Criteria

Articles that fulfilled the following criteria were selected for this study:

1. RCTs in humans with parallel group or crossover design.
2. The resveratrol treatment group received resveratrol-containing supplements, and the control group received a placebo at similar intervals.
3. Measuring mean changes in biological risk markers, such as total cholesterol (TC), triglyceride (TG), low-density lipoprotein cholesterol (LDL-C), high-density lipoprotein cholesterol (HDL-C), body weight, body mass index (BMI), waist circumference (WC), hemoglobin A1c (HbA1c), HOMA index, insulin, leptin, fasting glucose, fat percentage, and adiponectin level.
4. Data were presented as mean (±standard deviation, SD) or with 95% confidence intervals (95% CI) for the placebo and intervention groups.
5. Involving human RCTs published in the English language.

Reviews, conference abstracts, and studies with unavailable full text were excluded.

**FIGURE 1:**
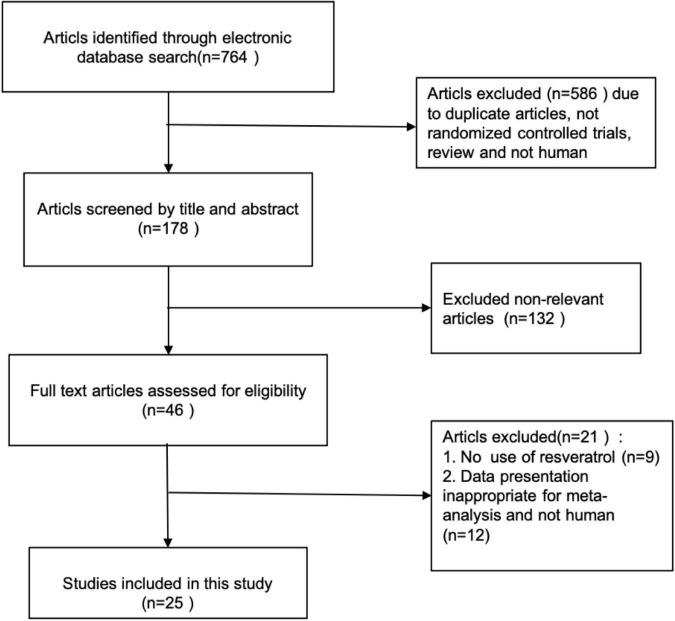
Literature search and review flowchart for selection of studies.

The process of study selection is shown in the PRISMA flow chart (Figure 1).

### Data Extraction

Two researchers independently performed study selection and extracted data of included studies using an Excel form. Disagreement between both researchers was resolved by consensus. The following items were extracted: first author’s name, publication year, location, age and gender, sample size (intervention and placebo groups), duration of intervention, number of sessions (or dose), underlying diseases, and mean value and standard deviation (SD) in intervention and placebo groups for TC, TG, LDL-C, HDL-C, body weight, BMI, WC, HbA1c, HOMA index, insulin, leptin, fasting glucose, fat percentage, and adiponectin level.

### Quality Assessment of Studies

The methodological quality and bias of all eligible studies were assessed by two independent reviewers using the Cochrane Collaboration risk of bias tool and standard Excel forms Any ambiguity or discrepancy in this course was resolved by discussion and involvement of a third person. Using the following seven criteria, we assessed the quality of studies: (1) random sequence generation, (2) allocation concealment, (3) blinding of participants and personnel, (4) blinding of outcome assessment, (5) incomplete outcome data, (6) selective reporting, and (7) other probable sources of risk biases.

### Data Synthesis

Patient characteristics are summarized in detail and are shown in Table 1. The authors estimated clinical information of all eligible studies on anthropometric measurements including: (1) TC, (2) TG, (3) LDL-C, (4) HDL-C, (5) body weight, (6) BMI, (7) WC, (8) HbA1c, (9) HOMA index, (10) insulin, (11) leptin, (12) fasting glucose, (13) fat percentage, and (14) adiponectin level. For calculating pooled effects, continuous data were pooled using mean difference (MD) with 95% confidence interval (CI). Inverse variance with a random-effect was applied. All related statistical analyses were performed with the Rev Man V.5.3 and STATA V.15 software. Cochran’s *Q*- test and the *I*^2^ statistic were performed to test for heterogeneity and quantify the proportion of total variation that resulted from heterogeneity (Higgins and Thompson, 2002), and *P* < 0.05 was regarded as significant heterogeneity (Melsen et al., 2014).

**TABLE 1:** Characteristics of included studies.

Authors	Publication year	Sample size		Population/Country	Intervention (daily dose)	Duration	Age (y)	
		Control	Intervention				Control	Intervention
Witte et al., 2014	2014	23	23	Germany	200 mg	26 weeks	64.8 ± 6.8	63.7 ± 5.3
Movahed et al., 2020	2020	13	13	Iran	1000 mg	60 days	23.61 ± 6.67	
Mansur et al., 2017	2017	24	24	Brazil	500 mg	1 months	58.46 ± 3.44	
Hoseini et al., 2019	2019	28	28	Iran	500 mg	4 weeks	63.3 ± 10.1	61.0 ± 8.6
Apostolidou et al., 2015	2015	18	15	Greece	150 mg	4 weeks	50.4 ± 14.1	38.2 ± 13.1
Faghihzadeh et al., 2015	2015	25	25	Iran	500 mg	12 weeks	46.28 ± 9.52	44.0 ± 10.10
Batista-Jorge et al., 2020	2020	9	12	Brazil	250 mg	3 months	30–60	
Imamura et al., 2017	2017	25	25	Japan	100 mg	12 weeks	58.2 ± 10.1	57.4 ± 10.6
Bhatt et al., 2012	2012	29	28	India	250 mg	3 months	57.7 ± 8.71	56.6 ± 8.91
Yoshino et al., 2012	2012	14	15	United States	120 mg	12 weeks	59.8 ± 4.3	58.2 ± 4.0
Zortea et al., 2016	2016	9	10	Brazil	200 mg	1 months	41.00 ± 7.87	46.40 ± 11.18
Kantartzis et al., 2018	2018	52	53	Germany	150 mg	12 weeks	18–70	
Simental-Mendía and Guerrero-Romero, 2019	2019	36	35	Mexican	100 mg	3 months	20–65	
Arzola-Paniagua et al., 2016	2016	24	15	Mexico	100 mg	24 weeks	38.8 ± 9.59	33.7 ± 11.9
de Ligt et al., 2020	2020	21	20	Netherlands	150 mg	6 months	62 ± 1.5	61 ± 1.3
Poulsen et al., 2013	2013	12	12	Denmark	150 mg	4 weeks	31.96 ± 2.9	44.76 ± 3.5
Macedo et al., 2015	2015	30	30	Brazil	100 mg	3 months	22.3 ± 1.78	21.4 ± 1.77
van der Made et al., 2015	2015	45	45	Netherlands	150 mg	4 weeks	60 ± 7	
Seyyedebrahimi et al., 2018	2018	23	23	Iran	800 mg	2 months	58.7 ± 6.06	54.96 ± 6.37
Chen et al., 2015	2015	30	30	China	300mg	3 months	20–60	
Timmers et al., 2011	2011	11	11	Netherlands	50 mg	1 months	52.5 ± 2.1	52.5 ± 2.1
Asghari et al., 2018	2018	30	30	Iran	600mg	12 weeks	39.27 ± 5.51	39.80 ± 7.74
Thazhath et al., 2016	2016	14	14	Australia	500mg	4 weeks	67.5 ± 1.6	
Kjær et al., 2017	2017	24	21	Denmark	150 mg	16 weeks	47.8 ± 1.30	
Kjær et al., 2017	2017	24	21	Denmark	1000 mg	16 weeks	47.8 ± 1.30	

We conducted pre-planned subgroup and sensitivity analyses for all outcomes based on clinical variables available to investigate the potential sources of heterogeneity. Subgroup analyses were performed to evaluate the source of heterogeneity of RSV supplementation, including duration of intervention, type of intervention, type of disease, type of intervention, baseline body weight, and BMI. Sensitivity analyses were conducted by removing one study at a time in order to assess the robustness of the results. Publication bias was evaluated using Begg’s statistics, with *P*-values < 0.05 considered statistically significant (Sterne et al., 2001).

## Results

### Search Results

A total of 764 citations were initially identified by electronic database searches. Twenty-five articles were incorporated into the final meta-analysis after removal of duplicates by checking titles and abstracts and excluding non-relevant articles. The process of study selection of relevant RCTs with more details is shown in the PRISMA flow chart (Figure 1). All the 25 articles were placebo-controlled randomized controlled trials (RCTs). 21 articles were of parallel group design and 4 of cross-over design. The selected articles had a total of 1,171 participants, including 578 in the placebo group and 593 in the intervention group. The number of participants in each group is ranged from 9 to 53. The included articles were published from inception to October 2021 in our current meta-analysis. The clinical data extracted from the 25 articles were measured using specific enzymatic ELISA kits (American Diabets Association, 2017), including serum concentrations of TC, TG, HDL-C, LDL-C, adiponectin, leptin, insulin, and fasting glucose. The characteristics of selected RCTs, with more details, are summarized in Table 1.

**FIGURE 2:**
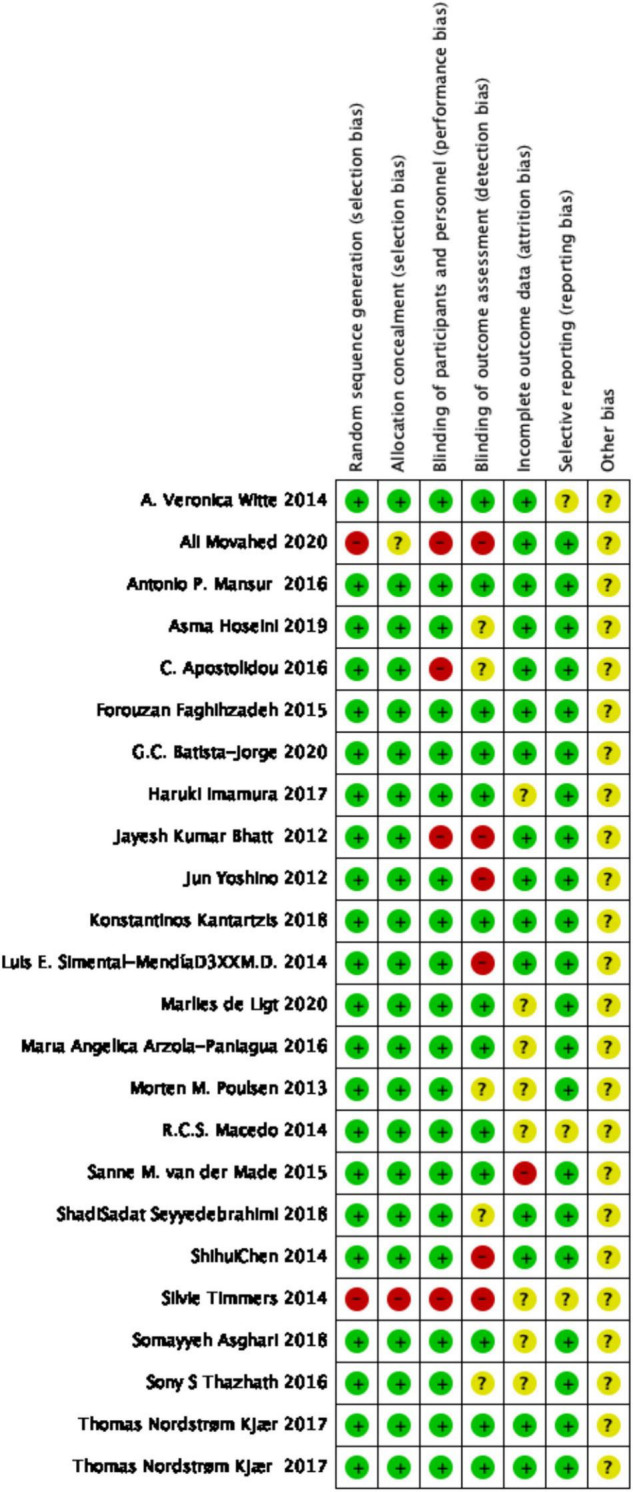
The methodological quality of included studies (risk of bias).

The methodological quality and bias of all the eligible studies were assessed using the Cochrane Collaboration risk of bias tool and standard Excel forms. The risk-of-bias summary of review authors’ judgments on each item for each included study is presented in Figure 2. Details on findings indicated that 8 studies had low bias risk, 19 studies had unclear bias risk, and 9 studies had high bias risk.

### Main Outcomes

#### Effects on Metabolism Indicators

**FIGURE 3:**
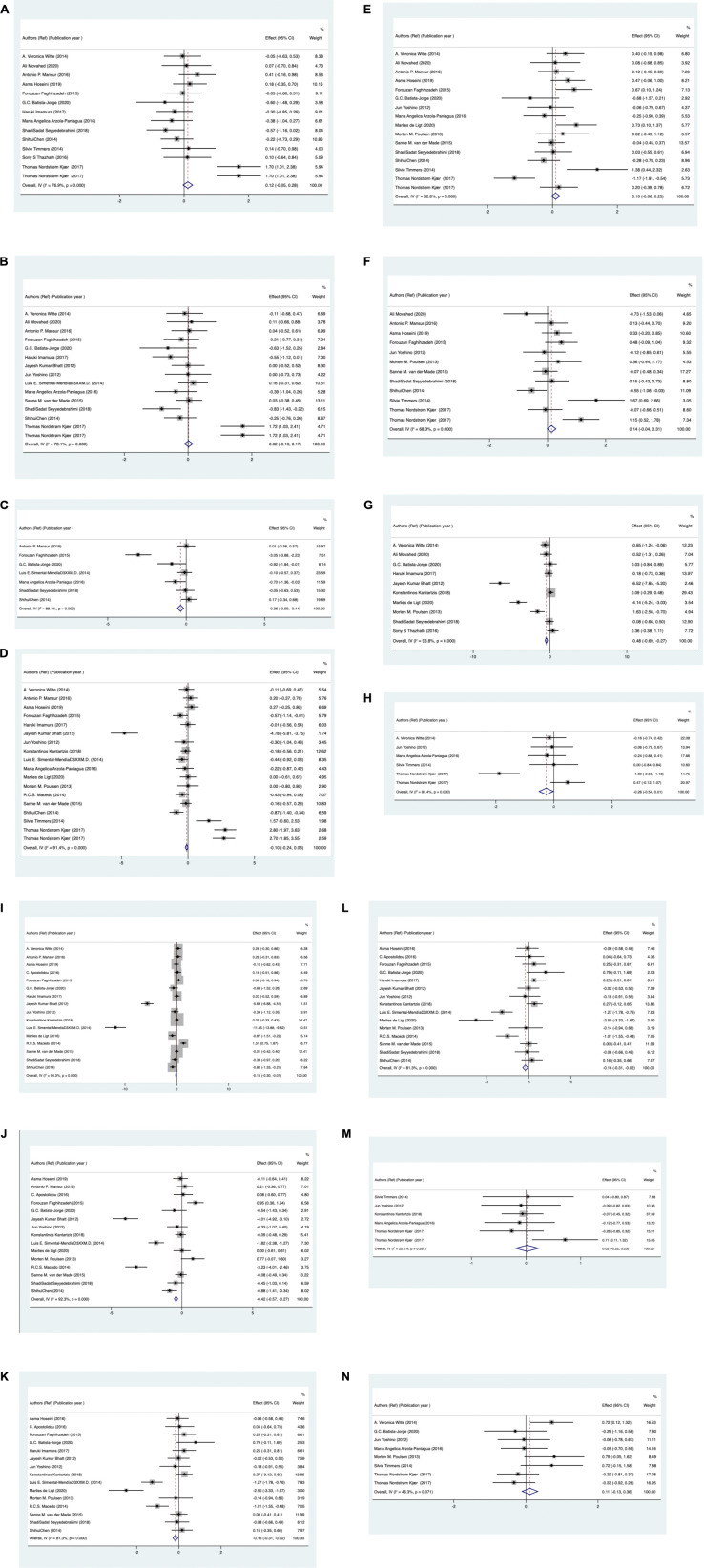
Standardized mean difference of metabolic markers meta-analysis for **(A)** body weight, **(B)** for body mass index, **(C)** for waist circumference, **(D)** fasting glucose, **(E)** insulin, **(F)** HOMA index, **(G)** HbA1c, **(H)** fat percentage, **(I)** total cholesterol, **(J)** low density lipoprotein cholesterol, **(K)** high density lipoprotein cholesterol, **(L)** triglyceride, **(M)** for adiponectin, **(N)** for leptin levels in resveratrol and control groups.

Effects of RSV on metabolism indicators are presented in Figure 3. A random effects model was used to pool the results. There was a significant decrease following RSV administration in WC with an SMD of –0.36 (95% CI: –0.59 to –0.14; *P* = 0.002; *I*^2^ = 88%), hemoglobin A1c (HbA1c): –0.48 (–0.69 to –0.2; *P* ≤ 0.001; *I*^2^ = 94%), TC: –0.15 (–0.3 to –0.01; *P* = 0.003; *I*^2^ = 94%), low-density lipoprotein cholesterol (LDL-C): –0.42 (–0.57 to –0.27; *P* ≤ 0.001; *I*^2^ = 92%), and HDL-C:0.16 (–0.31 to –0.02; *P* = 0.03; *I*^2^ = 81%). Meanwhile, we found that RSV had no significant effect on body weight with an SMD of.12 (–0.05 to 0.28; *P* = 0.18; *I*^2^ = 76%), BMI: 0.02 (–0.13 to 0.17; *P* = 0.83; *I*^2^ = 78%), fasting glucose: –0.10 (–0.24 to 0.03; *P* = 0.14; *I*^2^ = 91%), insulin:0.1 (–0.06 to 0.25; *P* = 0.42; *I*^2^ = 62%), HOMA index: 0.14 (0.04 to 0.31; *P* = 0.13; *I*^2^ = 68%), fat percentage: –0.26 (–0.54 to 0.01; *P* = 0.06; *I*^2^ = 80%), triglyceride (TG): 0.06 (–0.12 to.23; *P* = 0.53; *I*^2^ = 82%), adiponectin:0.02 (–0.22 to 0.25; *P* = 0.89; *I*^2^ = 22%), and leptin:0.11 (–0.13 to 0.36; *P* = 0.38; *I*^2^ = 46%).

#### Subgroup and Sensitivity Analyses

We found that heterogeneity was significantly high from prior comparisons. Subsequently, subgroup analyses were conducted to assess the possible source of heterogeneity, and the results are detailed in Table 2. In our current meta-analysis, some subgroups did not complete the analysis because of under-representation in number of trials. More detailed analyses were performed on subgroups with representation number of trials. WC was significantly decreased in obese patients (SMD: –0.39; 95% CI: –0.78, 0). According to BMI, our results exhibited a higher significantly decreased in patients with BMI < 30 kg/m^2^ (0.75; 95% CI: –1.29, –0.21) vs. BMI ≥ 30 kg/m^2^ (–0.27; –0.52, –0.02). Regarding interval of intervention, our current meta-analysis indicated that RSV supplementation significantly reduced WC in trials with intervention interval of 9-16 weeks (–0.73; –1.12, –0.34) compared with trails with duration < 8 weeks (–0.05; 0.36, 0.25). Considering the different dosages of RSV used in trials, we found that 200–500 mg/day (–0.49; –0.82, –0.17) for dose showed more significant reduction in WC than the dose of 200 mg/day (–0.29; –0.67,0.09). There are approximately similar significant findings in fasting glucose, HbA1c, TC, HDL-C, and LDL-C based on potential moderator variables. Subgroup analysis of different grouping types produced significantly different results.

**TABLE 2:** Subgroup analysis.

Variables		Number of SMD included	Subgroup	Pooled SMD (random effect)	95% CI	*I*^2^(%)	Overall *I*^2^ (%)
BW	Type of disease	4	Obesity	–0.02	[–0.38, 0.33]	38	76.9
		7	Other	0.23	[0.00, 0.46]	87	
		3	T2DM	–0.01	[–0.35, 0.33]	0	
	BMI groups	5	BMI ≥ 30 kg/m2	0.6	[0.28, 0.93]	89	
		9	BMI < 30 kg/m2	–0.06	[–0.25, 0.14]	0	
	Dosage of resveratrol (mg/day)	5	<200 mg resveratrol	0.14	[–0.14, 0.43]	83	
		6	200–500	0.02	[–0.22, 0.26]	0	
		3	>500 mg resveratrol	0.3	[–0.08, 0.69]	92	
	Duration of study (week)	4	<8 weeks	0.23	[–0.09, 0.54]	0	
		8	9–16 weeks	0.14	[–0.08, 0.36]	86	
		2	≥17 weeks	–0.19	[–0.63, 0.24]	0	
BMI	Type of disease	5	Obesity	–0.09	[–0.36, 0.17]	0	78.1
		8	Other	0.16	[–0.04, 0.37]	87	
		2	T2DM	–0.25	[–0.63, 0.13]	48	
	BMI groups	4	BMI ≥ 30 kg/m2	0.69	[0.33, 1.05]	91	
		11	BMI < 30 kg/m2	–0.13	[–0.29, 0.04]	4	
	Dosage of resveratrol (mg/day)	7	<200 mg resveratrol	0.07	[–0.14, 0.28]	78	
		5	200–500	–0.15	[–0.41, 0.11]	0	
		3	>500 mg resveratrol	0.23	[–0.16, 0.62]	93	
	Duration of study (week)	4	<8 weeks	–0.07	[–0.32, 0.17]	58	
		9	9–16 weeks	0.14	[–0.07, 0.34]	84	
		2	≥17 weeks	–0.23	[–0.66, 0.21]	0	
WC	Type of disease	3	Obesity	–0.39	[–0.78, –0.00]	47	88.4
		4	Other	–0.33	[–0.61, –0.06]	93	
		0	T2DM				
	BMI groups	5	BMI ≥ 30 kg/m2	–0.27	[–0.52, –0.02]	91	
		2	BMI < 30 kg/m2	–0.75	[–1.29, –0.21]	0	
	Dosage of resveratrol (mg/day)	2	<200 mg resveratrol	–0.29	[–0.67, 0.09]	50	
		4	200–500	–0.49	[–0.82, –0.17]	93	
		1	>500 mg resveratrol				
	Duration of study (week)	3	<8 weeks	–0.05	[–0.36, 0.25]	0	
		3	9–16 weeks	-0.73	[–1.12, –0.34]	95	
		1	≥17 weeks				
Fasting glucose	Type of disease	9	Obesity	–0.1	[–0.28, 0.09]	45	91.4
		6	Other	0.04	[–0.20, 0.29]	95	
		3	T2DM	–0.44	[–0.80, –0.08]	97	
	BMI groups	9	BMI ≥ 30 kg/m2	–0.34	[–0.49, –0.18]	87	
		6	BMI < 30 kg/m2	0.85	[0.54, 1.15]	93	
	Dosage of resveratrol (mg/day)	12	<200 mg resveratrol	–0.05	[–0.21, 0.11]	82	
		5	200–500	–0.53	[–0.80, –0.27]	95	
		1	>500 mg resveratrol				
	Duration of study (week)	6	<8 weeks	0.02	[–0.21, 0.24]	66	
		9	9–16 weeks	–0.19	[–0.39, 0.00]	95	
		3	≥17 weeks	–0.1	[–0.45, 0.25]	0	
Insulin	Type of disease	8	Obesity	0.05	[–0.18, 0.29]	88	62.8
		7	Other	0	[–0.22, 0.23]	71	
		1	T2DM	0.47	[–0.06, 1.00]		
	BMI groups	7	BMI ≥30 kg/m2	–0.11	[–0.39, 0.18]	92	0
		9	BMI < 30 kg/m2	0.14	[–0.05, 0.32]	11	
	Dosage of resveratrol (mg/day)	8	<200 mg resveratrol	–0.01	[–0.24, 0.21]	90	
		5	200–500	0.15	[–0.11, 0.40]	61	
		3	>500 mg resveratrol	0.1	[–0.26, 0.47]	0	
	Duration of study (week)	7	<8 weeks	0.2	[–0.02, 0.43]	28	
		6	9–16 weeks	–0.15	[–0.40, 0.11]	75	
		3	≥17 weeks	0.15	[–0.25, 0.55]	96	
HOMA index	Type of disease	5	Obesity	0.15	[–0.13, 0.42]	61	68.3
		6	Other	0.08	[–0.16, 0.33]	77	
		1	T2DM	0.32	[–0.20, 0.85]		
	BMI groups	8	BMI ≥ 30 kg/m2	–0.01	[–0.21, 0.19]	42	
		4	BMI < 30 kg/m2	0.6	[0.25, 0.96]	75	
	Dosage of resveratrol (mg/day)	5	<200 mg resveratrol	0.1	[–0.17, 0.38]	63	
		4	200–500	0.07	[–0.20, 0.35]	63	
		3	>500 mg resveratrol	0.3	[–0.07, 0.68]	85	
	Duration of study (week)	7	<8 weeks	0.14	[–0.09, 0.36]	59	
		5	9–16 weeks	0.13	[–0.14, 0.40]	78	
		0	≥17 weeks				
HbA1c	Type of disease	4	Obesity	–0.44	[–0.75, –0.12]	95	93.8
		4	Other	–0.24	[–0.57, 0.09]	30	
		2	T2DM	–1.1	[–1.62, –0.59]	99	
	BMI groups	7	BMI ≥ 30 kg/m2	–0.29	[–0.52, –0.07]	93	
		3	BMI < 30 kg/m2	–1.54	[–2.09, –0.99]	94	
	Dosage of resveratrol (mg/day)	5	<200 mg resveratrol	–0.46	[–0.72, –0.20]	93	
		3	200–500	–0.81	[–1.33, –0.28]	98	
		2	>500 mg resveratrol	–0.23	[–0.70, 0.23]	0	
	Duration of study (week)	4	<8 weeks	–0.3	[–0.66, 0.07]	72	
		4	9–16 weeks	–0.3	[–0.58, –0.01]	97	
		2	≥17 weeks	–1.4	[–1.92, –0.88]	96	
TC	Type of disease	7	Obesity	0.06	[–0.14, 0.27]	80	94.3
		6	Other	–0.47	[–0.71, –0.22]	93	
		3	T2DM	–0.56	[–0.92, –0.19]	97	
	BMI groups	2	BMI ≥ 30 kg/m2	–0.76	[–1.27, –0.25]	0	
		14	BMI < 30 kg/m2	–0.17	[–0.32, –0.02]	93	
	Dosage of resveratrol (mg/day)	9	<200 mg resveratrol	–0.11	[–0.29, 0.07]	92	
		6	200–500	–0.39	[–0.64, –0.13]	94	
		1	>500 mg resveratrol	–0.38	[–0.96, 0.20]		
	Duration of study (week)	6	<8 weeks	–0.34	[–0.56, –0.11]	92	
		8	9–16 weeks	–0.11	[–0.31, 0.10]	94	
		2	≥17 weeks	–0.23	[–0.66, 0.20]	85	
TG	Type of disease	7	Obesity	0.3	[0.04, 0.57]	90	92.3
		3	Other	–0.21	[–0.54, 0.12]	0	
		3	T2DM	–0.06	[–0.37, 0.25]	0	
	BMI groups	4	BMI ≥ 30 kg/m2	–0.25	[–0.62, 0.12]	74	
		9	BMI < 30 kg/m2	0.14	[–0.05, 0.33]	85	
	Dosage of resveratrol (mg/day)	7	<200 mg resveratrol	0.3	[0.04, 0.55]	89	
		5	200–500	–0.13	[–0.38, 0.12]	21	
		1	>500 mg resveratrol	–0.24	[–0.82, 0.34]		
	Duration of study (week)	5	<8 weeks	0.08	[–0.20, 0.35]	0	
		6	9–16 weeks	0.16	[–0.09, 0.41]	91	
		2	≥17 weeks	–0.36	[–0.82, 0.10]	85	
HDL-C	Type of disease	7	Obesity	–0.22	[–0.43, –0.01]	87	81.3
		5	Other	–0.22	[–0.47, 0.03]	81	
		3	T2DM	0.05	[-0.26, 0.36]	0	
	BMI groups	3	BMI ≥ 30 kg/m2	–0.67	[–1.16, –0.19]	93	
		12	BMI < 30 kg/m2	–0.11	[–0.26, 0.04]	71	
	Dosage of resveratrol (mg/day)	9	<200 mg resveratrol	–0.32	[–0.50, –0.14]	87	
		5	200–500	0.13	[–0.12, 0.38]	0	
		1	>500 mg resveratrol	–0.08	[–0.66, 0.50]		
	Duration of study (week)	6	<8 weeks	–0.27	[–0.49, –0.05]	72	
		8	9–16 weeks	0.04	[–0.15, 0.23]	65	
		1	≥17 weeks	–2.45	[–3.28, –1.62]		
LDL-C	Type of disease	8	Obesity	–0.24	[–0.44, –0.04]	89	81.3
		5	Other	–0.49	[–0.75, –0.23]	92	
		2	T2DM	–1.06	[–1.52, –0.61]	98	
	BMI groups	3	BMI ≥ 30 kg/m2	0.07	[–0.36, 0.50]	53	
		12	BMI < 30 kg/m2	–0.48	[–0.64, –0.32]	93	
	Dosage of resveratrol (mg/day)	8	<200 mg resveratrol	–0.45	[–0.65, –0.25]	92	
		6	200–500	–0.35	[–0.60, –0.10]	94	
		1	>500 mg resveratrol	–0.44	[–1.03, 0.15]		
	Duration of study (week)	7	<8 weeks	–0.27	[–0.48, –0.06]	85	
		7	9–16 weeks	–0.64	[–0.86, –0.41]	95	
		1	≥17 weeks	0	[–0.61, 0.61]		

For HbA1c level, there was a significant reduction in type 2 diabetes mellitus (T2DM) group (–1.1; –1.62, –0.59) compared with obese patients (–0.44; –0.75, –0.12), and other underlying diseases (0.24; –0.57, 0.09). Considering BMI groups, our results exhibited a higher significant decrease in patients with BMI < 30 kg/m^2^ (–1.54; –2.09, –0.99) vs. BMI ≥ 30 kg/m^2^ (–0.29; –0.52, –0.07). Regarding duration of intervention, there was a significant reduction in trials with intervention intervals > 17 weeks (–1.4; –1.92, –0.88) vs. interval < 8 weeks (–0.30; –0.66, 0.07) and 9-16 weeks (–0.3; –0.58, –0.01). As for dosage, there was a significant reduction in trials when dose of 200–500 mg/day (–0.81; –1.33, –0.28) vs. dose of 200 mg/day (–0.46; –0.72, –0.2).

For lipid metabolism, significant results were mainly focused on TC, HDL-C, and LDL-C. Regarding different types of disease, we found that RSV supplementation significantly decreased TC and LDL-C in patients with T2DM vs. obese patients and other diseases. However, for HDL-C, there was a significant reduction in obese patients vs. T2DM and other diseases. Considering BMI, we found that resveratrol supplementation significantly decreased TC and HDL-C in trials with BMI < 30 kg/m^2^ vs. BMI > 30 kg/m^2^. However, for LDL-C, BMI < 30 kg/m^2^ was significant vs. BMI > 30 kg/m^2^. According to duration of intervention, there was a significant reduction in TC and HDL-C with duration < 8 weeks vs. durations of intervention > 17 weeks and 9–16 weeks. For LDL-C, duration of intervention of 9-16 weeks is significant compared with durations < 8 weeks and >17 weeks. Considering dosage of RSV intake, there was a significant reduction in LDL-C and HDL-C when dose of 200 mg/day vs. doses of 200–500 mg/day and >500 mg/day. However, for TC, there was a significant reduction in trials when dose of 200–500 mg/day vs. doses of 200 mg/day.

According to our current meta-analysis, the subgroup analyses indicated that no significant differences were observed based on moderator variables BW, BMI, insulin, HOMA index, TG, adiponectin, leptin, and fat percentage. More details are presented in Table 2. In addition, the results for sensitivity-pooled SMD were not significant for BMI, insulin, HbA1c, LDL-C, TG, adiponectin, and leptin. The lower and higher pooled SMDs for these indicators in the sensitivity analysis with more details are presented in Table 3.

**TABLE 3:** Other indicators in the sensitivity analysis.

Indicators	Lower pooled SMD	Excluding study	Higher pooled SMD	Excluding study
BMI	–0.07 [–0.22, 0.09]	Kjær et al., 2017	0.07 [–0.08, 0.23]	Seyyedebrahimi et al., 2018
HbA1c	–0.70 [–0.95, –0.46]	Kantartzis et al., 2018	–0.32 [–0.53, –0.11]	Bhatt et al., 2012
LDL-C	–0.51 [–0.66, –0.35]	Faghihzadeh et al., 2015	–0.30 [–0.46, –0.15]	Simental-Mendía and Guerrero-Romero, 2019
TG	–0.11 [–0.29, 0.07]	Macedo et al., 2015	0.13 [–0.05, 0.31]	de Ligt et al., 2020
Adiponectin	–0.07 [–0.35, 0.21]	Kjær et al., 2017	0.07 [–0.23, 0.36]	Kantartzis et al., 2018
Leptin	–0.01 [–0.28, 0.26]	Witte et al., 2014	0.30 [0.00, 0.60]	Kjær et al., 2017
Insulin	–0.01 [–0.16, 0.15]	de Ligt et al., 2020	0.14 [–0.02, 0.30]	Arzola-Paniagua et al., 2016

#### Publication Bias and Quality Assessment

The Begg test to identify publication bias and funnel plots are shown in Supplementary Figure S1. Tests confirmed that publication bias was not significant for RSV on body weight (*P* = 0.87), BMI (*P* = 1), glucose (*P* = 0.256), insulin (*P* = 0.893), HOMA index (*P* = 0.373), fat percentage (*P* = 1), LDL-C (*P* = 0.235), HDL-C (*P* = 0.322), TG (*P* = 0.502), adiponectin (*P* = 0.26), and leptin (*P* = 0.536). Possible publication bias was detected for WC (*P* = 0.035), HbA1c (*P* = 0.012), and TC (*P* = 0.034). The Duval and Tweedie non-parametric/trim and fill method was used fill in missing theoretical studies. However, the pooled SMDs were not significantly changed in terms of TC, HbA1c, and WC after the Duval and Tweedie test.

## Discussion

The conclusions of the current meta-analysis demonstrated that RSV intake had a significant effect on WC, HbA1c, TC, LDL-C, and HDL-C reduction, but that it had no effect on leptin and adiponectin levels. The sensitivity analyses indicated that after exclusion of related RCTs, the pooled results of body weight, fasting glucose, and HOMA index had significant changes. In contrast, the finding has shown the opposite results for WC, TC, and HDL-C. The effects of modulating lipid by regulation of TC content, metabolic disturbances, and reduction in TC are thought to be exerted predominantly through LDL-C and HDL-C. For people with diabetes, our findings indicated that RSV intake can reduce glycosylated HbA1c and blood glucose, and exhibits long-term glycemic regulation. However, the effect of reducing serum glucose is believed to be not associated with decreased insulin resistance or increased serum insulin. To summarize, the findings indicated that RSV supplementation exerts anti-obesity effects by regulating glycolipid metabolism and improving metabolic disturbances.

Resveratrol (RSV) (3,5,4′-trihydroxy-*trans*-stilbene), a polyphenolic phytoalexin that exhibits diverse pharmacological actions, acts as a natural antioxidant (Roy et al., 2021) with a plethora of beneficial effects on reducing intracellular reactive oxygen species (ROS) and oxidative stress. Antioxidants are compounds that modulate the mechanisms of homeostasis of glucose and lipids by decreasing the levels of ROS (Hussain et al., 2016; Kattoor et al., 2017; Unuofin and Lebelo, 2020). A tremendous amount of animal and clinical studies had proved that RSV could improve chronic diseases, especially metabolic syndrome. Studies have suggested that RSV supplementation exerts effects on lipid by regulating genes involved in lipid metabolism (Ahn et al., 2008; Azorín-Ortuño et al., 2012), which is a potential complementary approach to prevent obesity. In rodents, RSV blunted high-fat diet (HFD)-induced hepatic lipid storage and metabolic disorders (Alberdi et al., 2013; Zhou et al., 2018), muscle lipid storage (Zhang Y. J. et al., 2019; Gong et al., 2020) and ameliorated insulin resistance (Jeon et al., 2012; Zhang Y. J. et al., 2019; Gong et al., 2020). In human trials, it was also observed that glucose and lipids were improved after RSV treatment (Bhatt et al., 2012; Hsu et al., 2020; Izquierdo et al., 2021; van Polanen et al., 2021), but our findings contradict the previous reports (Sahebkar, 2013; Sahebkar et al., 2015; Timmers et al., 2016; Kjær et al., 2017; de Ligt et al., 2018; Tabrizi et al., 2020). Discrepancies among existing bodies of evidence might be linked to insufficient sample size and characteristics existing in study design, such as study populations, statistical analyses, comorbid conditions, and dosages and formulations of RSV used.

Lipid metabolism played an important role in the physiological and pathological states, and disorder may lead to many pathophysiological consequences. Hyperlipidemia is a sign of lipid metabolism disorder, one of the most common chronic diseases, and clinical diabetes; it associated with comorbidities such as hypertension and obesity, also known as a major driver of many metabolic diseases with no effective treatment. Studies have found that body fat distribution is highly associated with altered lipid metabolism (Zhang et al., 2018). Regulation of hepatic lipid metabolism is an integral component of overall regulatory program to maintain whole body metabolic homeostasis (Warne et al., 2011). It has been shown that lipid metabolism also influences modulation of inflammation and cytokine secretion (Glass and Olefsky, 2012; Kumar et al., 2014). One of the most common tumors, ccRCC, is associated with dysfunctions in lipid metabolism, and the abnormal lipid accumulation phenomenon in ccRCC has been observed for a long time (Wettersten et al., 2017). A number of studies have reported the importance of lipid microdomains in protein sorting and transportation (Gruenberg, 2003). Metabolic disorders, such as insulin resistance and obesity, have a basis in dysregulated lipid metabolism (Sato et al., 2014), and abnormal lipid metabolism is the most intuitive and common. Host physiology and metabolic diseases are highly relevant to lipid homeostasis. The observed changes in lipid compositions potentially indicate altered expression of diseased states. In metabolically compromised humans, RSV induced remodeling of myocellular lipid stores (van Polanen et al., 2021), reduced of lipid accumulation, and increased glycogen storage in muscle and hepatic cells by promoting lipolysis in adipocytes (Gong et al., 2020).

In our current meta-analysis, we found that RSV supplementation exerts a beneficial effect on improving metabolic (lipid and glucose) disturbances. A previous study has suggested the role of RSV by regulating skeletal muscle, liver lipid, and energy metabolism (Serrano et al., 2021). The liver plays a unique central role in modulating lipid absorption and energy homeostasis (Mashek, 2013). Mechanistically, RSV could ameliorate NAFLD and hepatic steatosis in obese mice by promoting the SIRT1/AMPK pathway (Tian et al., 2016), possibly mediated by activating SIRT11 as a Sirt1 enhancer (Zhang et al., 2018), and ameliorating the accumulation of LDs by mediating a SIRT1/ATF6-dependent mechanism (Zhou et al., 2018). In skeletal muscle, RSV ameliorates high-fat diet-induced insulin resistance and fatty acid oxidation *via* the ATM-AMPK axis (Zhang Y. J. et al., 2019). Besides, other mechanisms have been proposed. For example, Zhang has suggested that the beneficial effect of RSV is related with change in the expression of several lipid metabolism-related miRNAs and genes (Zhang H. Z. et al., 2019), especially ssc-miR-181a, ssc-miR-370, ssc-miR-21, and ssc-miR-27a (Zhang H. Z. et al., 2019). Zhuang et al. (2021) proposed the effect of improving gut immune response and microbiota function. Furthermore, in the future, large-scale and well-designed trials will be warranted to confirm the mechanism of the therapeutic effect of RSV.

## Conclusion

Taken together, these results suggest that RSV has a dramatic impact on regulating lipid and glucose metabolism, and the major clinical value of resveratrol intake is for obese and diabetic patients. The efficacy of resveratrol supplementation in lipid metabolism was clarified in the results of the systematic review. We hope that this study could provide more options for clinicians using RSV.

## Data Availability Statement

The original contributions presented in the study are included in the article/Supplementary Material, further inquiries can be directed to the corresponding author/s.

## Author Contributions

QZ and YW conceptualized and designed the study, and drafted and reviewed the initial manuscript. XH and CZ defined concepts and search items, data extraction process, and methodological appraisal. QZ, YW, and SF planned the extraction of data and statistical analysis. QC and SF provided the critical insights. All authors approved and contributed to the final written manuscript.

## Conflict of Interest

The authors declare that the research was conducted in the absence of any commercial or financial relationships that could be construed as a potential conflict of interest.

## Publisher’s Note

All claims expressed in this article are solely those of the authors and do not necessarily represent those of their affiliated organizations, or those of the publisher, the editors and the reviewers. Any product that may be evaluated in this article, or claim that may be made by its manufacturer, is not guaranteed or endorsed by the publisher.

## Abbreviations

BW: body weight
BMI: body index mass index
WC: waist circumference
TC: total cholesterol
TG: triglyceride
LDL-C: low-density lipoprotein cholesterol
HDL-C: high-density lipoprotein cholesterol
HbA1c: hemoglobin A1c
T2DM: type two diabetes mellitus.
