# Corrigendum: Efficacy of resveratrol supplementation on glucose and lipid metabolism: A meta-analysis and systematic review

**DOI:** 10.3389/fphys.2022.918751

**Published:** 2022-07-22

**Authors:** Qian Zhou, Yanmei Wang, Xuke Han, Shunlian Fu, Chan Zhu, Qiu Chen

**Affiliations:** Hospital of Chengdu University of Traditional Chinese Medicine, Chengdu, China

**Keywords:** resveratrol, meta-analysis, lipid, T2DM, obese

In the published article, there was an error in the Correspondence section as author Qian Zhou was erroneously included as a **Corresponding Author**. The corrected Correspondence information appears below:

*Correspondence: Qiu Chen, chenqiu1005@cdutcm.edu.cn

The authors apologize for this error and state that this does not change the scientific conclusions of the article in any way. The original article has been updated.

