# Supplementary material for: Efficacy of Resveratrol Supplementation on Glucose and Lipid Metabolism: A Meta-Analysis and Systematic Review
Source: Front Physiol. 2022 Mar 31;13:795980. doi: 10.3389/fphys.2022.795980 (PMC9009313; doi:10.3389/fphys.2022.795980)
Supplement: Supplementary file 2 [file Image_1.pdf]

Figure S1. Funnel plot.

body weight

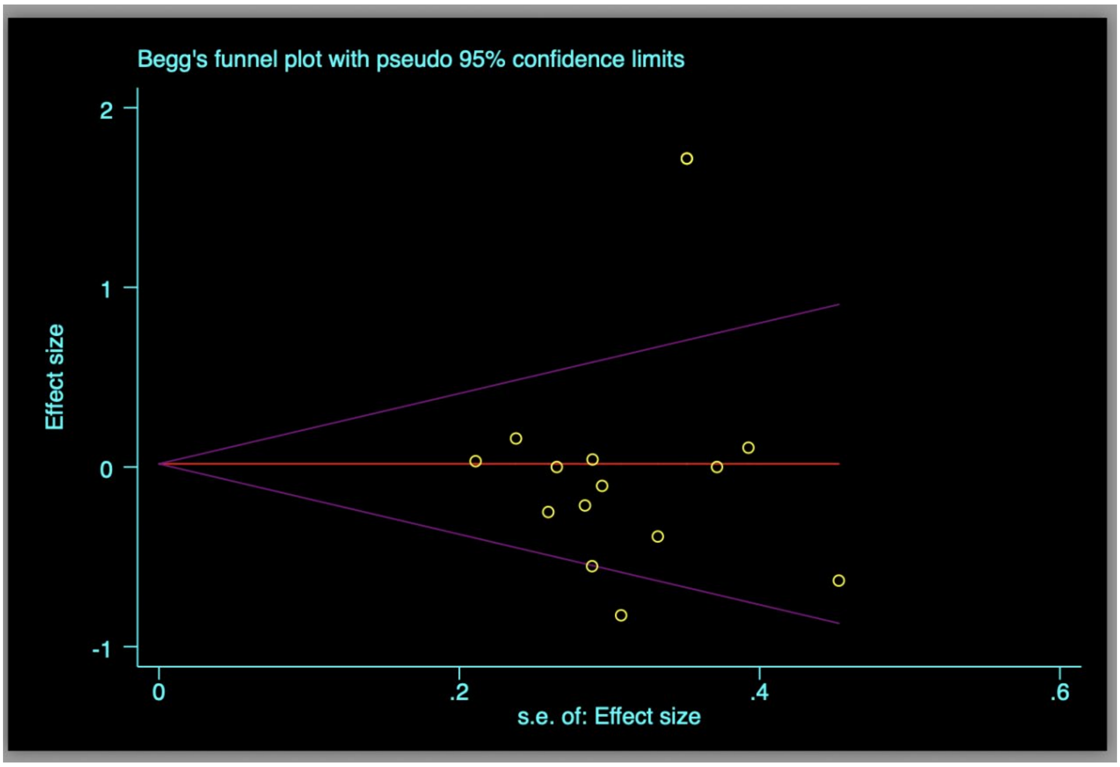

body mass index (BMI)

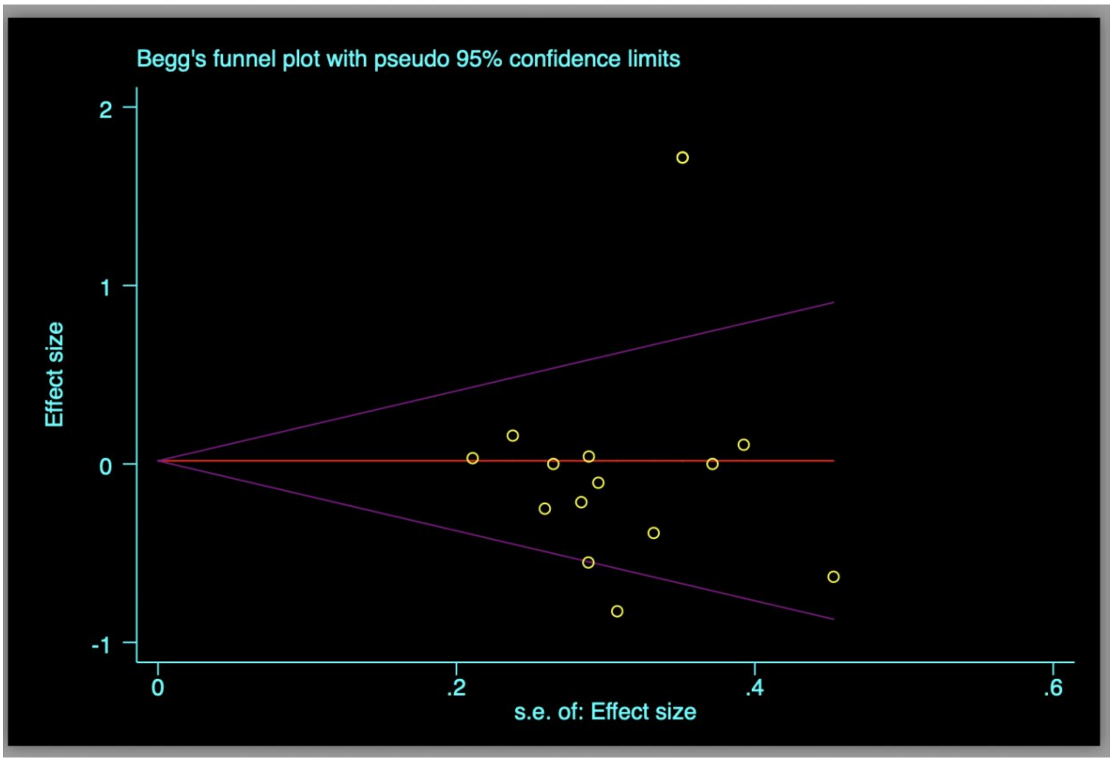

waist circumference

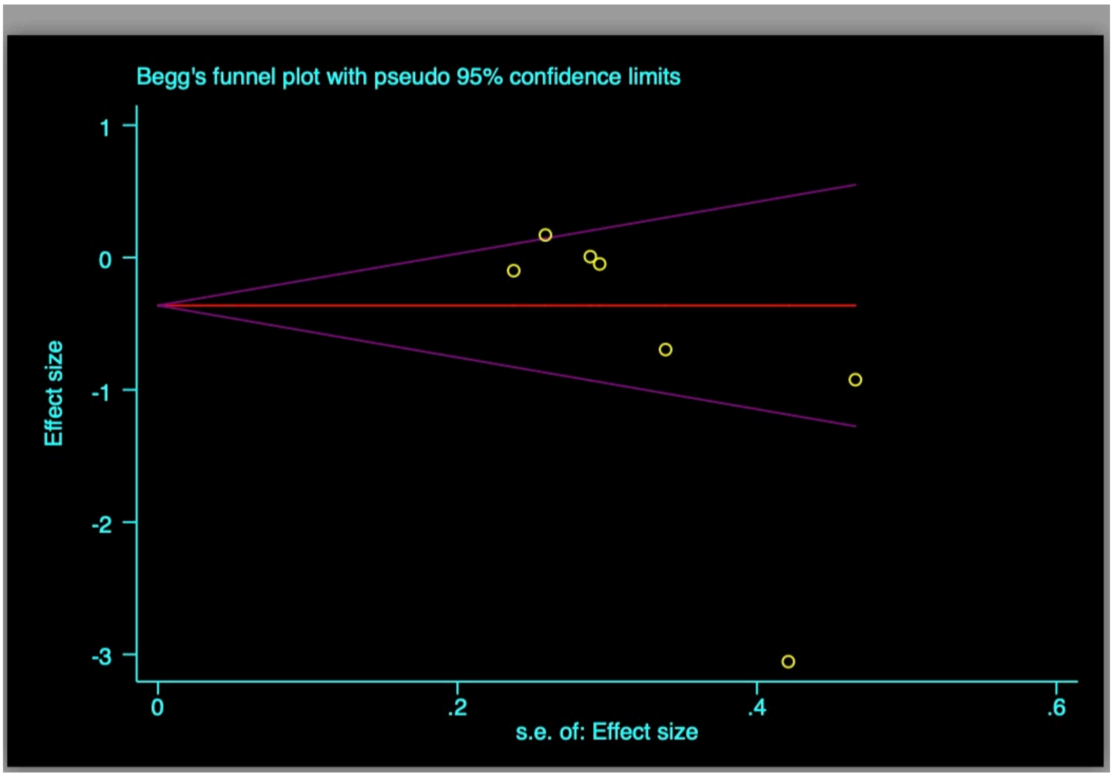

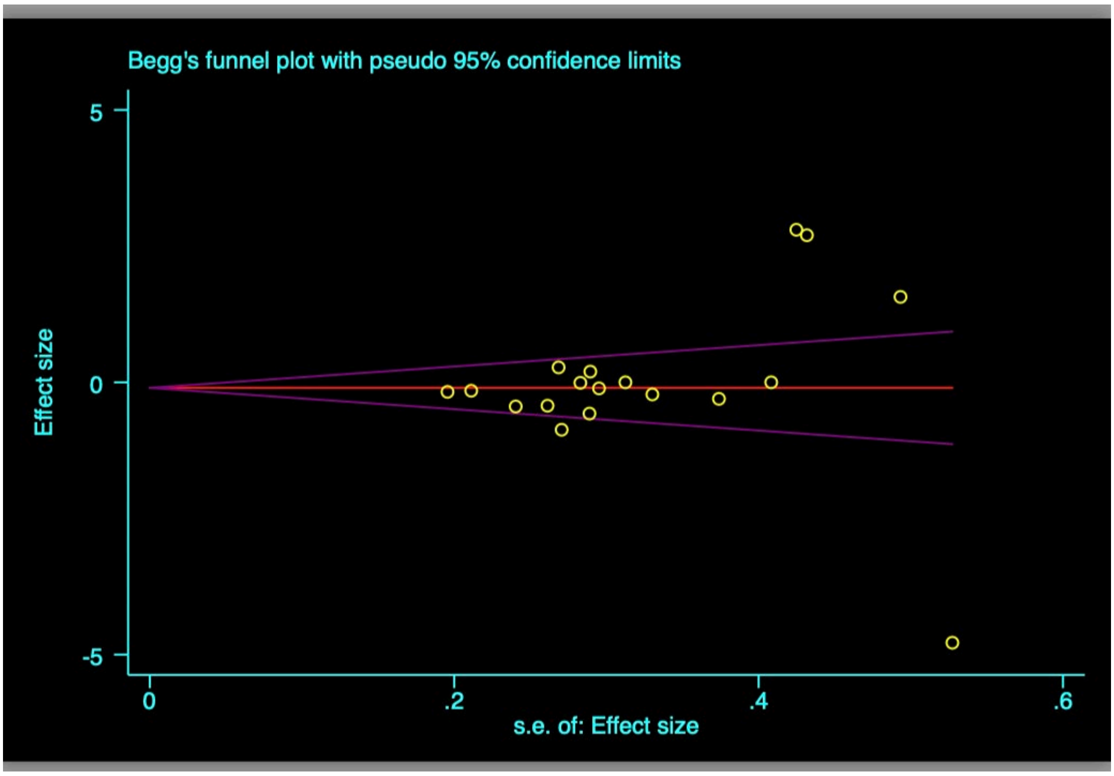

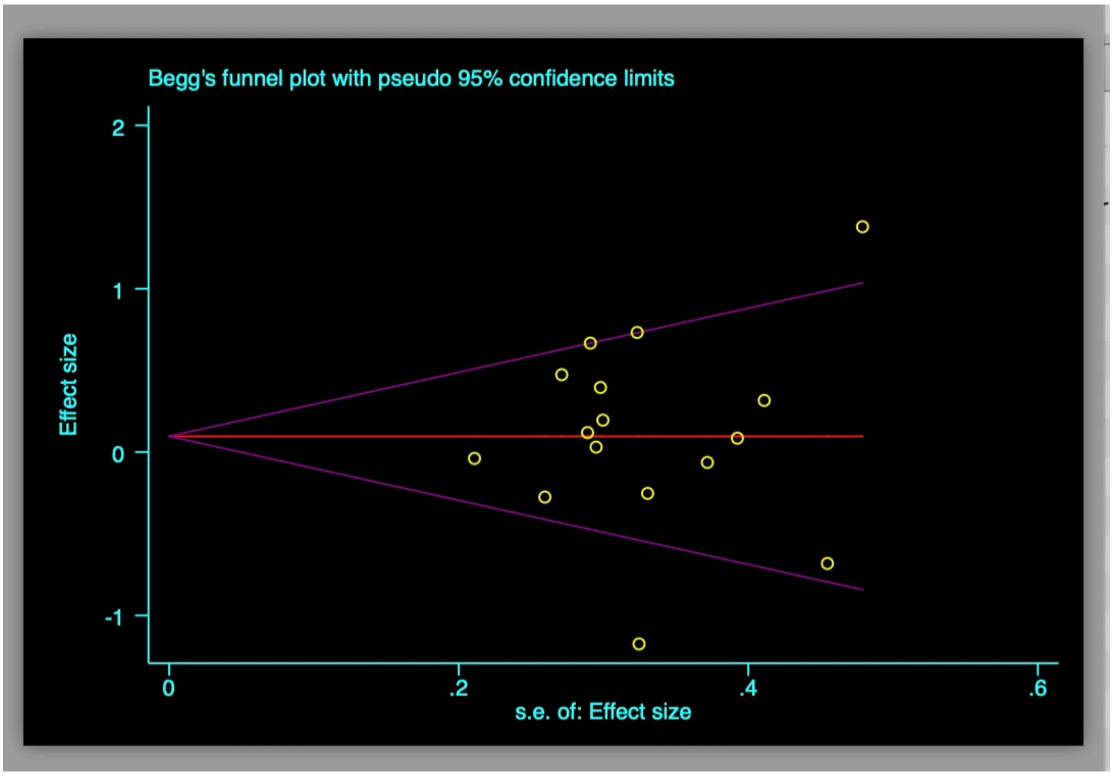
Begg's funnel plot with pseudo 95% confidence limits
Effect size
2
1
0
-1
0
.2
.4
.6
s.e. of: Effect size

HOMA index

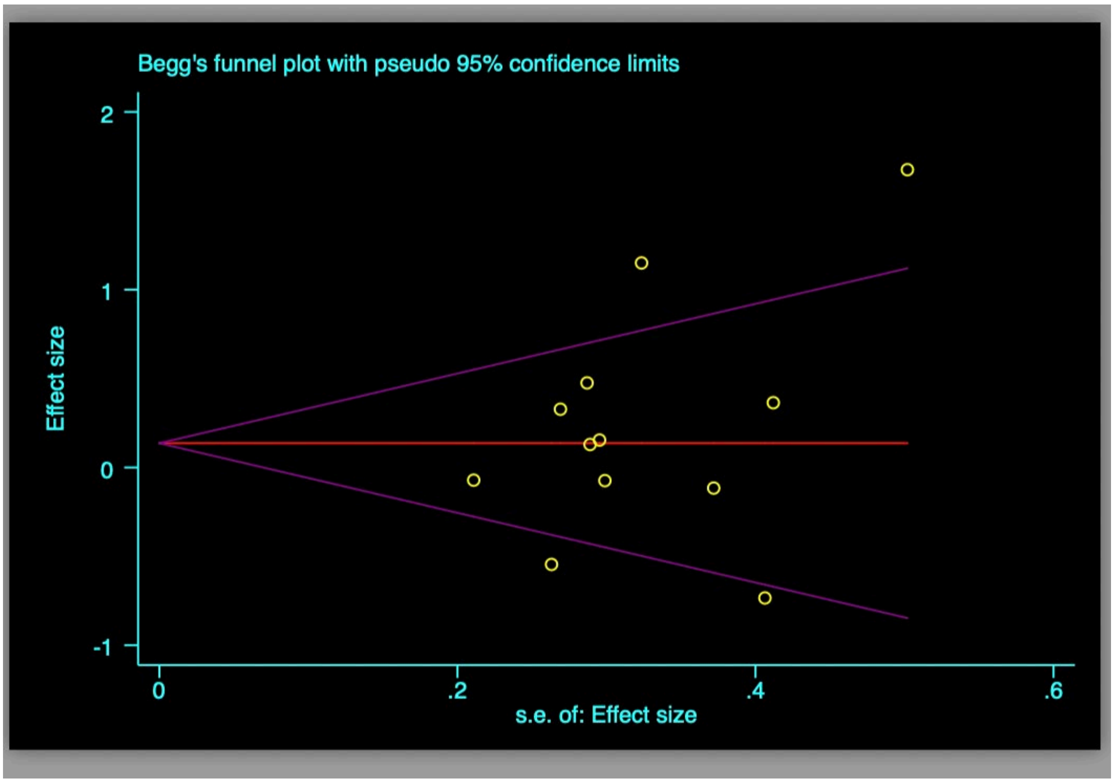
Begg's funnel plot with pseudo 95% confidence limits
Effect size
2
1
0
-1
0
.2
.4
.6
s.e. of: Effect size

HemoglobinA1c

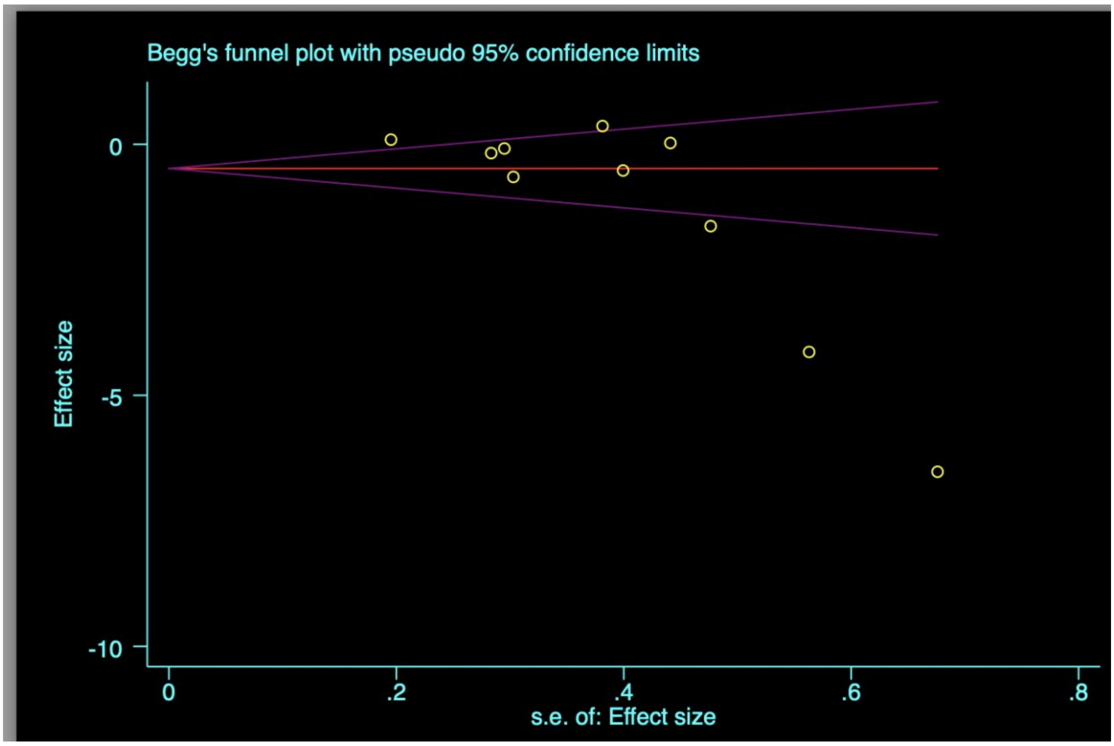

Fat percentage

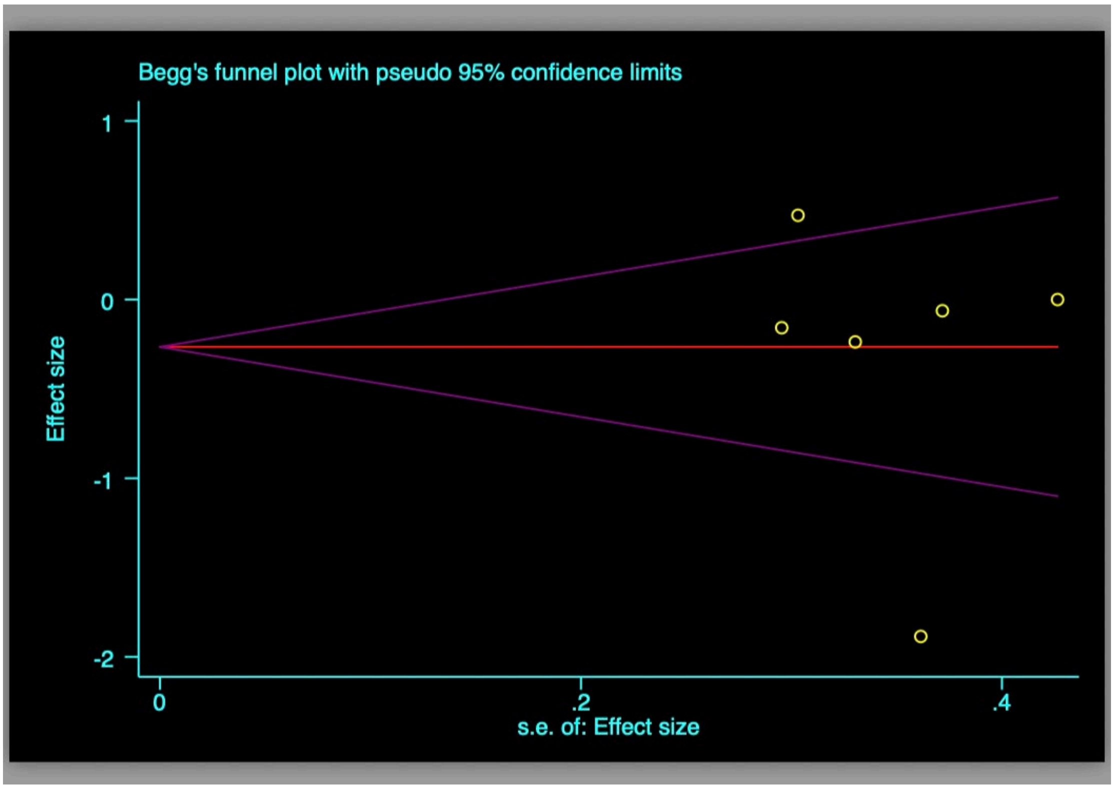

Total cholesterol

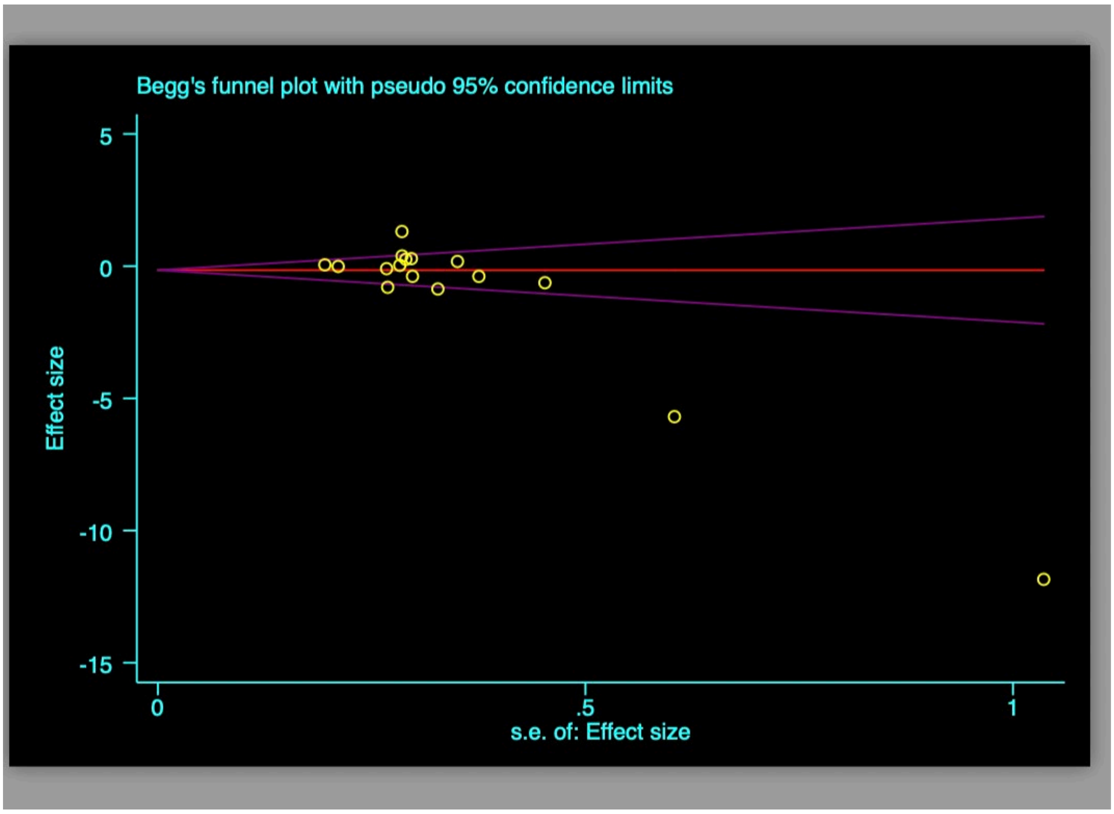

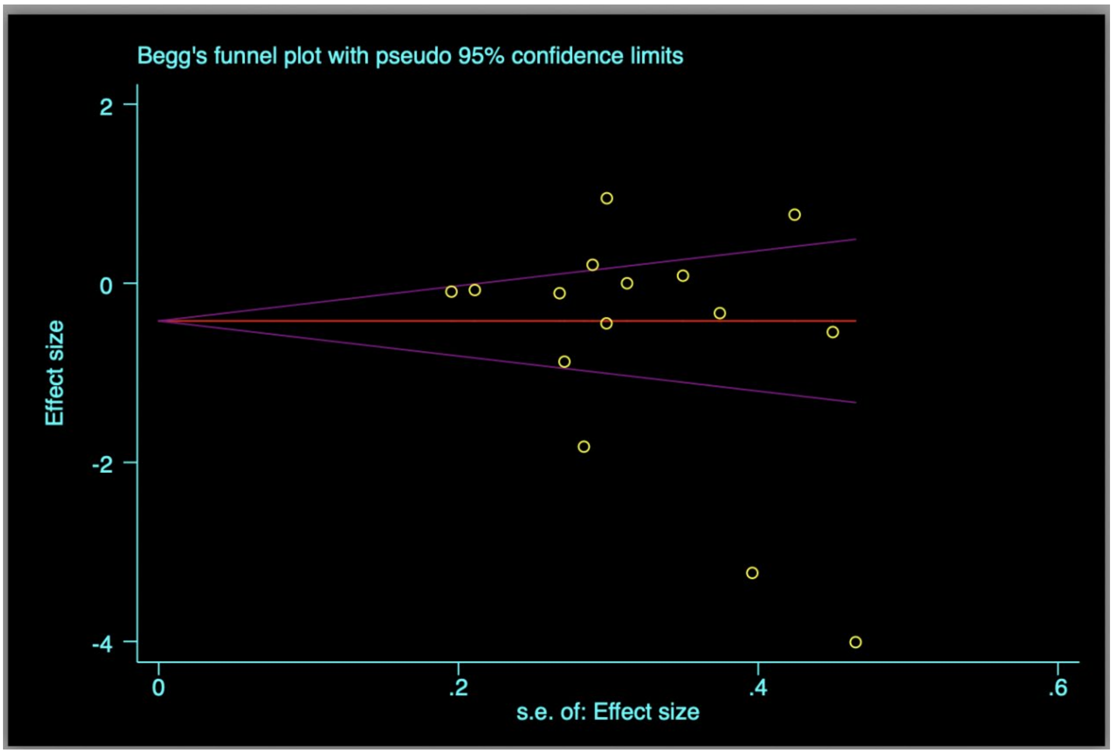

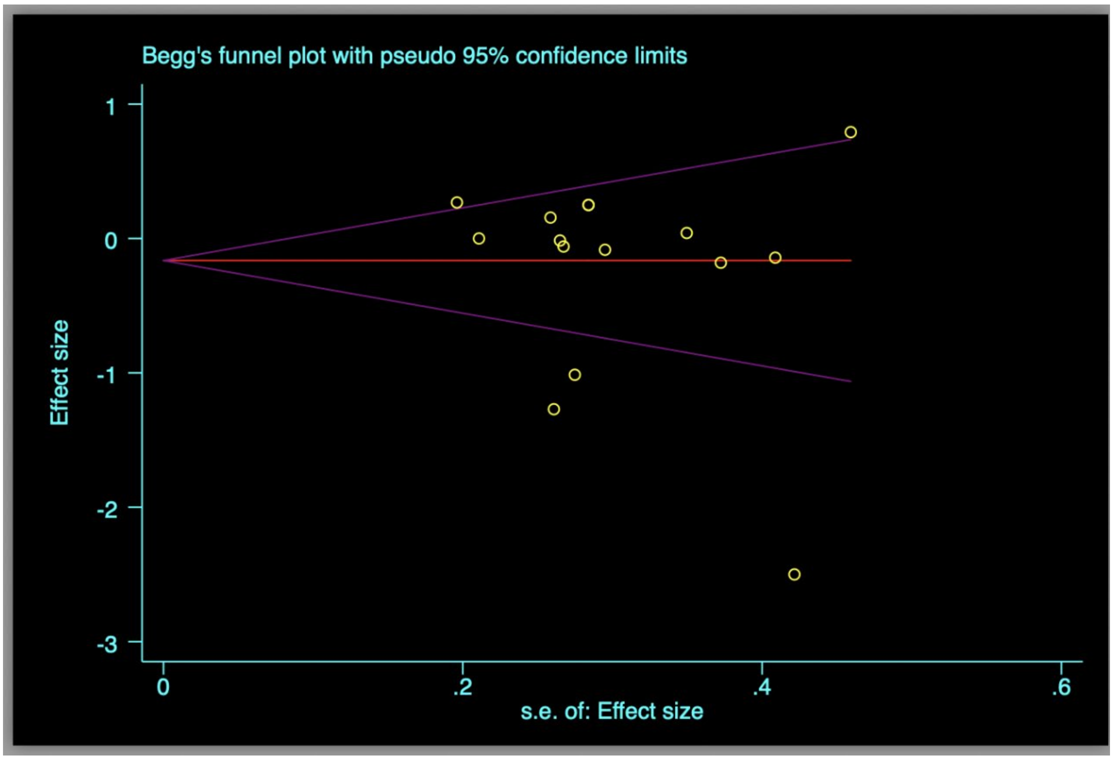

Triglycerides

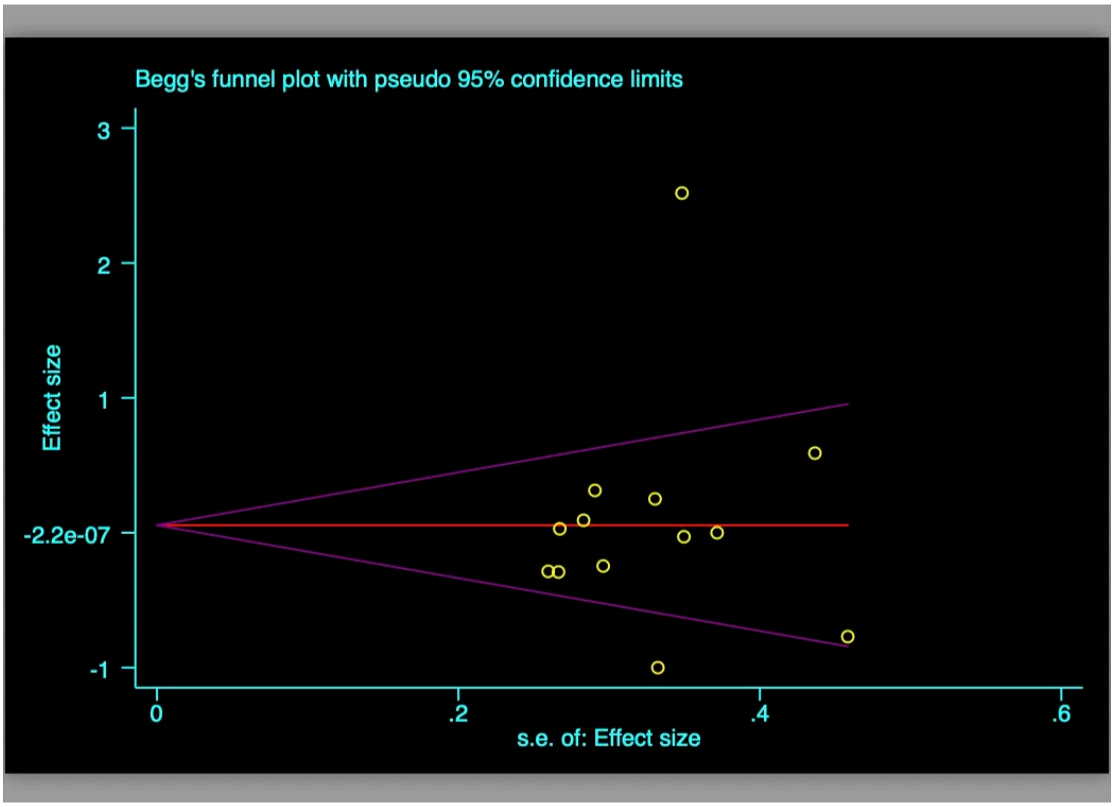

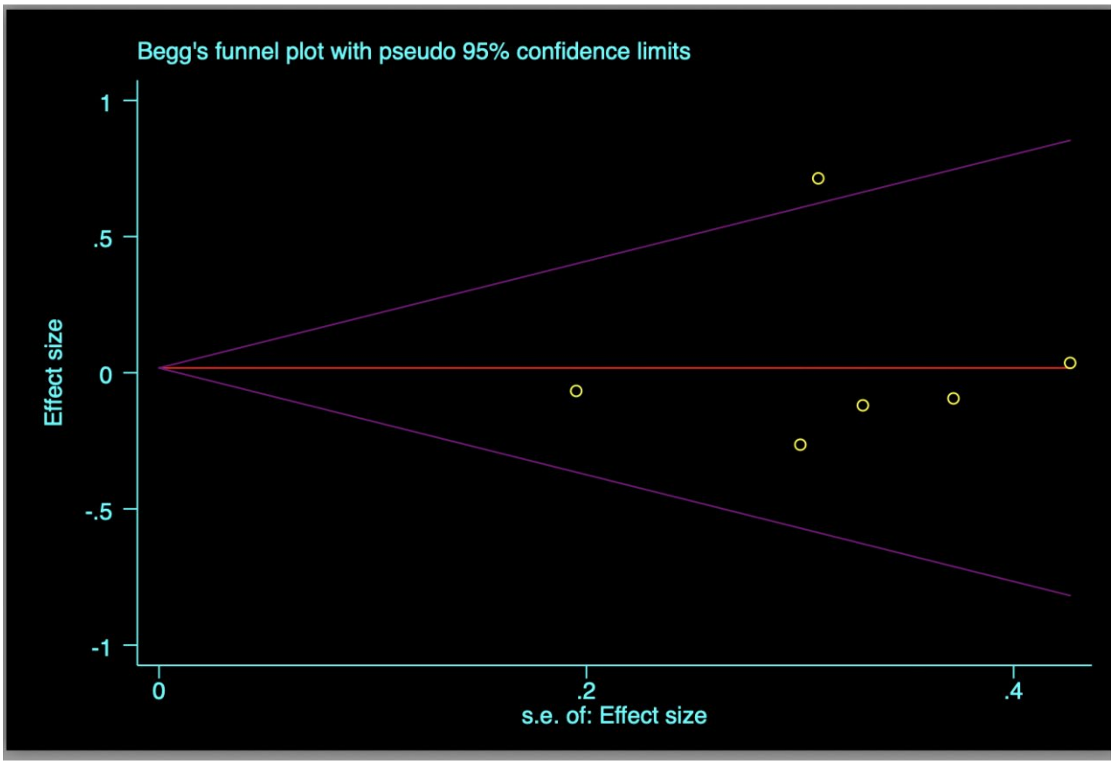
Begg's funnel plot with pseudo 95% confidence limits
Effect size
1
.5
0
-.5
-1
0
.2
.4
s.e. of: Effect size

Leptin

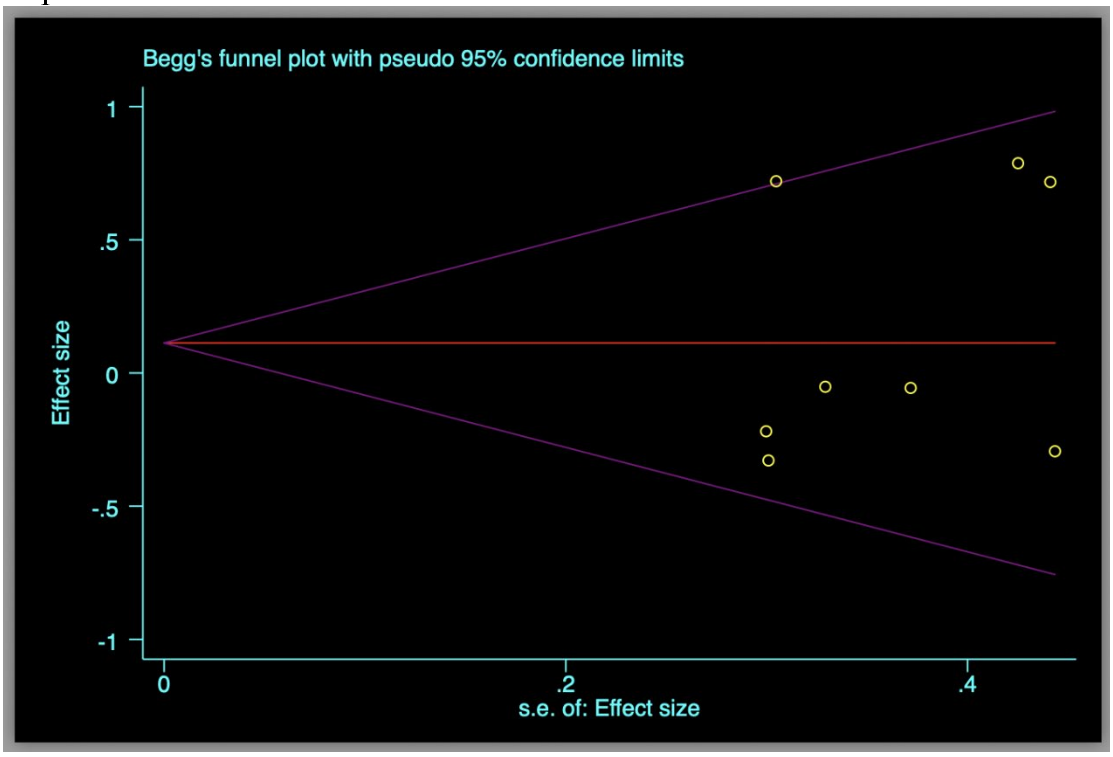
Begg's funnel plot with pseudo 95% confidence limits
Effect size
1
.5
0
-.5
-1
0
.2
.4
s.e. of: Effect size
